# Internet and Social Media For Health-Related Information and Communication in Health Care: Preferences of the Dutch General Population

**DOI:** 10.2196/jmir.2607

**Published:** 2013-10-02

**Authors:** Tom H Van de Belt, Lucien JLPG Engelen, Sivera AA Berben, Steven Teerenstra, Melvin Samsom, Lisette Schoonhoven

**Affiliations:** ^1^Radboud REshape and Innovation CenterRadboud University Medical CenterNijmegenNetherlands; ^2^Regional Emergency Healthcare NetworkRadboud University Medical CenterNijmegenNetherlands; ^3^Department for Health Evidencesection BiostatisticsRadboud University Medical CenterNijmegenNetherlands; ^4^Executive BoardRadboud University Medical CenterNijmegenNetherlands; ^5^Scientific Institute for Quality of HealthcareRadboud University Medical CenterNijmegenNetherlands; ^6^Faculty of Health SciencesUniversity of SouthamptonSouthamptonUnited Kingdom

**Keywords:** social media, patient participation, consumer health information, empowerment, Health 2.0

## Abstract

**Background:**

Health care is increasingly featured by the use of Web 2.0 communication and collaborative technologies that are reshaping the way patients and professionals interact. These technologies or tools can be used for a variety of purposes: to instantly debate issues, discover news, analyze research, network with peers, crowd-source information, seek support, and provide advice. Not all tools are implemented successfully; in many cases, the nonusage attrition rates are high. Little is known about the preferences of the Dutch general population regarding the use of the Internet and social media in health care.

**Objective:**

To determine the preferences of the general population in the Netherlands regarding the use of the Internet and social media in health care.

**Methods:**

A cross-sectional survey was disseminated via a popular Dutch online social network. Respondents were asked where they searched for health-related information, how they qualified the value of different sources, and their preferences regarding online communication with health care providers. Results were weighed for the Dutch population based on gender, age, and level of education using official statistics. Numbers and percentages or means and standard deviations were presented for different subgroups. One-way ANOVA was used to test for statistical differences.

**Results:**

The survey was completed by 635 respondents. The Internet was found to be the number one source for health-related information (82.7%), closely followed by information provided by health care professionals (71.1%). Approximately one-third (32.3%) of the Dutch population search for ratings of health care providers. The most popular information topics were side effects of medication (62.5%) and symptoms (59.7%). Approximately one-quarter of the Dutch population prefer to communicate with a health care provider via social media (25.4%), and 21.2% would like to communicate via a webcam.

**Conclusions:**

The Internet is the main source of health-related information for the Dutch population. One in 4 persons wants to communicate with their physician via social media channels and it is expected that this number will further increase. Health care providers should explore new ways of communicating online and should facilitate ways for patients to connect with them. Future research should aim at comparing different patient groups and diseases, describing best practices, and determining cost-effectiveness.

## Introduction

Health care is increasingly featured by the use of Web 2.0 communication and collaborative technologies that are reshaping the way patients and professionals interact [1]. This process, in which Web 2.0 tools are used in health care, is part of Health 2.0 (also known as Medicine 2.0) [2], an important fundament of which is the use of social media [3]. Kaplan and Haenlein [4] define social media as “a group of Internet-based applications that build on the ideological and technological foundations of Web 2.0 and that allow the creation and exchange of user-generated content.” Well-known examples are YouTube, Facebook, and Twitter. These can be used for a variety of purposes: to instantly debate issues, discover news, analyze research, network with peers, crowd-source information, seek support, and provide advice [5]. Research shows that larger health care organizations, such as hospitals, are increasingly using social media [6,7]. In many cases, the ultimate goal is to make health care better or more cost-efficient [8].

Since the arrival of social media interventions for health-related purposes, it has become clear that not all these interventions are actually successful. Although no studies exist that have investigated this problem for social media, eHealth literature, which overlaps with social media because both involve technology, could provide some insight into this problem. It is known that interventions are often not successful and/or the attrition rates may be high [9-11]. Several explanations for unsuccessful use have been described: (1) technology features (eg, imperfections of the technology), (2) inadequate reimbursement or legislation issues, (3) poor coordination and introduction of tools, and (4) personal characteristics of the intended use [9,12]. Personal characteristics seem to be particularly relevant because they concern the end-users of the tool. Examples of such characteristics, which are known to significantly influence use, are negative attitude toward technology, the extent to which a person feels he has the skills and expertise to be a competent caregiver, and age [9]. Therefore, determining the preferences or needs of potential users of tools is an important step in implementation [12-14]. Although studies have assessed patients’ preferences regarding the Internet in health care (eg, the preferred language on websites [15], the preferences of a Web-based intervention [16], preferences regarding social media and asthma patients [17], or the needs of elderly patients regarding eHealth [18]), less is known about the preferences or needs of consumers or the general public, especially regarding social media. A survey showed that 32% of all respondents (US adults) had used social media for health care purposes at one time or another [19]. Further insights, however, are lacking. Questions that arise in this context are: Where do people obtain online health-related information? Where do they connect with peers? Are they willing to ask their doctor questions using a webcam? And are there differences between different groups of the population (eg, by gender, age, or education)?

For that reason, we sought to determine the preferences of the general population in the Netherlands regarding the use of the Internet and social media in health care, by using an online survey that was disseminated via an online social network.

## Methods

### Design, Setting, and Population

A cross-sectional survey was disseminated via a popular Dutch online social network. Hyves was selected as the social network for dissemination of the survey. This social network has long been the most popular Dutch online social network, with 9.7 million members of all ages [20,21], comprising more than half of the Dutch population [22]. Hyves can be used to create a personal profile and connect with friends. Furthermore, users can like pages or create groups. Between October 4 and November 4, 2011, Hyves members aged at least 15 years were randomly invited through Hyves’ internal message system. There were no restrictions regarding sex, race, or income. The messages contained a description of the project (in Dutch) and a link to the survey.

### Questionnaire Development and Content

#### Overview

A first draft of the questionnaire was created by TB and subsequently discussed with LE and LS. This version was shared with 3 experts: a social media expert, a researcher (SB), and an epidemiologist. After discussion, consensus was reached and the survey was finalized and uploaded to the online system. The questionnaire consisted of 17 multiple-choice questions divided over 3 sections: (1) sociodemographic, (2) health-related information and Internet, and (3) respondents’ preferences regarding communication in health care. All questions were written in Dutch. The final survey (English version) is available in Multimedia Appendix 1.

#### Sociodemographic Section

The sociodemographic section contained questions about age, gender, and level of education.

#### Health-Related Information and Internet

In the health-related information and Internet section, respondents were asked where they searched for health-related information and how they qualified the value of different sources. The topics were:

1. Sources of health-related information;
2. Type of online information that is searched for;
3. Frequency of health-related searches; and
4. Perceived reliability of different sources.

#### Respondents’ Preferences Regarding Communication in Health Care

In the preferences section, preferences regarding communication in health care were acknowledged.

### Response

A total of 4232 people selected the link to the online survey, of which 679 filled out the survey. After excluding incomplete surveys or surveys completed by respondents under 15 years (n=44), 635 cases were analyzed. The mean response time was 6.13 minutes (SD 2.95).

### Statistical Analysis

The data were downloaded from the online system and analyzed in SPSS version 20 (IBM Corp, Armonk, NY, USA). We used descriptive statistics to examine the proportions for different age, gender, and education groups. Proportions for age were summarized in 6 age groups: 15-24, 25-34, 35-44, 45-54, 55-64, and 65 or older.

Answers regarding health-related information and the Internet as well as preferences of communication in health care were extrapolated to the Dutch population based on gender, age, and level of education. We decided to create 2 age groups based on different generations described in the literature [23]. The first group consisted of people aged 15-34 years. This group has been described as Generation Y and consists of people who grew up with the Internet. The second age group consisted of persons aged 35 years or older, including the Generation X and the so-called baby-boomers. Two levels of education were recognized. The first group consisted of people with no education or lower education, whereas the second group consisted of moderately or highly educated people.

For each stratum (combination of gender, age, and educational level), the response within the survey was estimated. The response of the stratum was then weighted by the relative frequency of that stratum within the Dutch population of 2011, acquired via Statistics Netherlands (Centraal Bureau voor de Statistiek, CBS) [22]. CBS is a Dutch governmental institution and part of the Dutch Ministry of Economic Affairs that is responsible for gathering and publishing official statistics about the Netherlands. CBS statistics are only published if they are valid and if the overall quality can be guaranteed. The following example shows how we weighed data: if the percentage of young males and old males saying yes was 40% and 60%, respectively, then this would result in a mean of 50% in our sample. Given that young and old males (from CBS statistics) form 0.3 and 0.7 of the Dutch male population, respectively, the percentage of males who would say yes in the Dutch population was estimated to be (0.3×40%) + (0.7×60%) = 54%.

We present numbers and percentages or means and standard deviations. To properly test differences between groups in the response (eg, male vs female) extrapolated to the Dutch population, we needed to take into account that (1) the precision of the estimated response percentages in strata is determined by the size of the strata in the survey, and (2) these response percentages are weighted by the relative frequency of those strata in the Dutch population. To accomplish this, we used the SPSS procedure 1-way ANOVA to (1) estimate the response percentages with their corresponding precision from the survey, and (2) perform the weighting by specifying the relative frequencies in contrast tests. Because the size of the strata was reasonably large (>25) and the response within strata was not close to zero or 100%, the ANOVA means and standard errors were considered a good approximation of the response percentages of the strata. *P* values <.05 were considered statistically significant.

## Results

### Sociodemographic

In total, 635 respondents completed the survey, consisting of 95 (15.0%) men and 540 (85.0%) women. Table 1 shows the age distribution for all respondents in 10-year age ranges. In all, 181 respondents (28.5%) had no education or low education and 454 (71.5%) were moderately or highly educated.

### Sources of Health-Related Information

Table 2 shows the popularity of different sources of health-related information estimated for the Dutch population. Internet and physicians were found to be the most popular sources (82.7% and 71.1%, respectively). Family and friends were mentioned by 20.5% of the Dutch population. People aged ≤ 34 years consulted their family and friends significantly more often than people older than 34 years (38.1% vs 13.5%, 1-way ANOVA, contrast test *t*_627_=3.52, *P*<.001). Higher educated people also consulted their family and friends more often (12.5% for lower educated people vs 24.7% for higher educated persons, 1-way ANOVA, contrast test *t*_627_=–2.05, *P*=.04). Patient information leaflets or books were the least popular information source (14.6%).

### Type of Online Information Searched For

The most popular information topics that were searched online (Table 3) were side effects of medication and symptoms (62.5% and 59.7%, respectively). People aged 35 years or older searched significantly more often for information on side effects than people younger than 35 years (68.7% vs 46.8%, 1-way ANOVA, contrast test *t*_627_=–2.63, *P*=.01). People younger than 35 years searched more often for symptoms than persons aged 35 or older (76.1% vs 53.2%, 1-way ANOVA, contrast test *t*_627_=2.65, *P*=.01). Furthermore, women indicated that they searched more often for information on diagnoses than men (58.8% vs 31.5%, 1-way ANOVA, contrast test *t*_627_=–4.13, *P*<.001).

**Table 1:** Survey respondents (N=635).

Subgroup		n (%)
**Gender**		
	Male	95 (15.0)
	Female	540 (85.0)
**Age**		
	15 - 24	74 (11.7)
	25 - 34	90 (14.2)
	35 - 44	144 (22.7)
	45 - 54	172 (27.1)
	55 - 64	129 (20.3)
	65 or older	26 (4.1)
**Education**		
	No/lower education	181 (28.5)
	Moderate or high education	454 (71.5)

**Table 2:** Sources for health-related information.

Subgroup		Total, %^a^	Group 1, %^b^	Group 2, %^b^	*t*_627_	*P*
**Gender**						
	Internet	82.7	82.8	82.6	0.11	.91
	Physician	71.1	66.2	74.9	–1.45	.15
	Family/friends	20.5	19.7	21.2	–0.29	.78
	Patient information (leaflets, books)	14.6	11.6	17.5	–1.19	.23
**Age**						
	Internet	82.7	87.4	74.0	0.99	.32
	Physician	71.1	63.8	74.0	–1.30	.19
	Family/friends	20.5	38.1	13.5	3.52	<.001
	Patient information (leaflets, books)	14.6	15.0	14.5	0.04	.97
**Education**						
	Internet	82.7	78.0	85.2	–1.25	.21
	Physician	71.1	69.8	71.8	–0.34	.74
	Family/friends	20.5	12.5	24.7	–2.05	.04
	Patient information (leaflets, books)	14.6	9.3	17.5	–1.43	.15

^a^Estimations for Dutch population (%) based on the study sample of 635 respondents. Note that these estimates are weighted sums of the cell response percentages; therefore, n’s cannot be provided (see Methods) for these percentages.

^b^For gender, group 1=male, group 2=female; for age, group 1=age ≤34 years, group 2=age>34 years; for education, group 1=no or low education, group 2=moderate or high education.

**Table 3:** Type of health-related information searched for online.

Subgroup		Total, %^a^	Group 1, %^b^	Group 2, %^b^	*t*_627_	*P*
**Gender**						
	Side effects medication	62.5	58.2	66.9	–1.17	.24
	Symptoms	59.7	58.7	60.5	–0.13	.90
	Diagnoses	45.6	31.5	58.8	–4.13	<.001
	Patients’ experiences	41.7	37.1	46.0	–1.27	.20
	Health care insurance	41.6	38.0	44.9	–0.99	.32
	Therapy	39.3	34.6	43.6	–1.35	.18
	My hospital	35.4	38.9	32.1	1.2	.23
	Ratings of health care providers	32.3	36.6	28.3	1.4	.16
	Health problems	14.8	13.8	15.7	–0.20	.83
	Manufacturers of medication	8.9	10.7	7.2	0.98	.33
	Second opinion	6.8	5.8	7.8	–0.65	.52
**Age**						
	Side effects medication	62.5	46.8	68.7	–2.63	.01
	Symptoms	59.7	76.1	53.2	2.65	.01
	Diagnoses	45.6	44.4	46.1	–0.21	.83
	Patients’ experiences	41.7	35.0	44.3	–1.1	.27
	Health care insurance	41.6	34.6	44.3	–1.11	.27
	Therapy	39.3	30.3	42.8	–1.43	.15
	My hospital	35.4	32.2	36.7	–0.55	.58
	Ratings of health care providers	32.3	30.2	33.2	–0.37	.71
	Health problems	14.8	10.6	16.4	–0.95	.34
	Manufacturers of medication	8.9	6.2	9.7	–0.65	.52
	Second opinion	6.8	10.0	5.6	1.01	.31
**Education**						
	Side effects medication	62.5	58.4	64.7	–0.86	.39
	Symptoms	59.7	55.2	62.0	–0.88	.38
	Diagnoses	45.6	40.9	48.1	–0.98	.33
	Patients’ experiences	41.7	36.4	44.6	–1.08	.28
	Health care insurance	41.6	31.7	46.9	–1.94	.05
	Therapy	39.3	33.6	42.3	–1.08	.28
	My hospital	35.4	29.0	38.8	–1.34	.18
	Ratings of health care providers	32.3	31.1	31.9	0.17	.86
	Health problems	14.8	12.8	15.9	–0.54	.59
	Manufacturers of medication	8.9	5.6	10.7	–1.25	.21
	Second opinion	6.8	4.3	8.2	–1.03	.30

^a^Estimations for Dutch population (%) based on the study sample of 635 respondents. Note that these estimates are weighted sums of the cell response percentages; therefore, n’s cannot be provided (see Methods) for these percentages.

^b^For gender, group 1=male, group 2=female; for age, group 1=age ≤34 years, group 2=age>34 years; for education, group 1=no or low education, group 2=moderate or high education.

### Frequency of Health-Related Searches

We determined the frequency of online health-related searches extrapolated to the Dutch population. In all, 92.0% indicated that they searched for health-related information at least once a year and 24.4% searched for health-related information at least every month.

Table 4 shows the search behavior of Dutch people before consulting a physician (eg, general practitioner or specialist). In all, 42.3% indicated that they sometimes searched online for health-related information and 18.4% indicated that they never searched online for information before visiting a physician. Table 4 also shows the search behavior after visiting a physician (general practitioner or specialist). In all, 44.4% indicated that they sometimes searched online for health-related information after visiting their physician and 17.0% indicated that they never searched online for information after having visited their physician.

### Perceived Reliability of Sources and Other Preferences

Table 5 shows the perceived reliability of sources of health-related information. On a scale from 1 (very unreliable) to 10 (very reliable), people rated their physician and their personal opinion as most reliable (7.3 and 7.5, respectively). Internet and family/friends scored 6.0 and 5.9 on the scale of reliability, respectively. The least reliable source is information retrieved via social media: 3.8 of 10. Family/friends were found to be more reliable by younger persons than older ones (6.7 vs 5.6, 1-way ANOVA, contrast test *t*_627_=3.29, *P*=.001). Furthermore, higher educated people rated their personal opinion as more reliable than lower educated persons did (7.7 vs 7.0, 1-way ANOVA, contrast test *t*_627_=–2.35, *P*=.02).

### Respondents’ Preferences Regarding Communication in Health Care

Table 6 shows to which extent Dutch people would like to communicate using social media or webcams. In all, 25.4% prefer to communicate with their health care provider via social media. Furthermore, 21.2% would like to communicate with their health care providers via a webcam. No statistical differences were found between subgroups.

**Table 4:** Online searches for health-related information before and after visiting physician (general practitioner or specialist).

Moment of search (before/after)		Total^a^	Gender^a^				Age^a^				Education^a^			
			Male	Female	*t*_627_	*P*	≤ 34	> 34	*t*_627_	*P*	No/low	Mod/high	*t*_627_	*P*
**Search before, %**					–1.69	.09			3.34	.001			1.65	.10
	Very often	4.3	4.1	4.5			11.0	1.7			2.9	5.1		
	Often	18.3	18.4	18.2			29.0	14.0			25.4	14.5		
	Sometimes	42.3	34.8	49.3			39.9	43.3			50.4	37.9		
	Rarely	16.7	18.5	15.0			9.3	19.6			8.7	21.0		
	Never	18.4	24.1	13.1			10.9	21.4			12.6	21.6		
**Search after, %**					–3.52	<.001			–0.88	.38			0.48	.63
	Very often	2.5	1.5	3.5			3.9	2.0			2.7	2.4		
	Often	14.8	10.9	18.5			12.8	15.6			10.8	17.0		
	Sometimes	44.4	36.6	51.8			36.6	47.5			53.3	39.6		
	Rarely	21.2	29.5	13.4			27.3	18.7			22.1	20.7		
	Never	17.0	21.5	12.8			19.4	16.1			11.1	20.2		

^a^Estimations for Dutch population (%) based on the study sample of 635 respondents. Note that these estimates are weighted sums of the cell response percentages; therefore, n’s cannot be provided (see Methods) for these percentages.

**Table 5:** Perceived reliability of sources for health-related information.

Subgroup		Total, mean^a^	Group 1, mean^b^	Group 2, mean^b^	*t*_627_	*P*
**Gender**						
	Self	7.5	7.4	7.5	–0.42	.67
	Physician	7.3	7.4	7.2	0.87	.38
	Internet	6.0	6.0	6.0	0.18	.86
	Friends/family	5.9	6.0	5.8	0.98	.33
	Social media	3.8	3.7	3.8	0.04	.97
**Age**						
	Self	7.5	7.8	7.3	1.47	.14
	Physician	7.3	7.6	7.2	1.12	.26
	Internet	6.0	6.2	5.9	0.97	.33
	Friends/family	5.9	6.7	5.6	3.29	.001
	Social media	3.8	3.2	4.0	–2.03	.04
**Education**						
	Self	7.5	7.0	7.7	–2.35	.02
	Physician	7.3	7.1	7.4	–0.77	.44
	Internet	6.0	6.0	6.0	–0.31	.76
	Friends/family	5.9	6.1	5.8	1.07	.29
	Social media	3.8	3.6	3.8	–0.69	.49

^a^Estimations for Dutch population (on a scale from 1 to 10; 1=very unreliable, 10=very reliable), based on the study sample of 635 respondents. Note that these estimates are weighted sums of the cell response numbers; therefore, n’s cannot be provided (see Methods) for these scores.

^b^For gender, group 1=male, group 2=female; for age, group 1=age ≤34 years, group 2=age>34 years; for education, group 1=no or low education, group 2=moderate or high education.

**Table 6:** Preferences for communication in health care.

Subgroup		Total, %^a^	Group 1, %^b^	Group 2, %^b^	*t* (df)	*P*
**Gender**						
	Would like to ask questions to health care provider via social media^a^	25.4	27.4	23.7	0.64 (573)	.52
	Would like to communicate with health care provider via webcam^c^	21.2	25.2	17.5	1.41 (563)	.16
**Age**						
	Would like to ask questions to health care provider via social media^a^	25.4	19.6	27.8	–1.04 (573)	.30
	Would like to communicate with health care provider via webcam^c^	21.2	11.7	25.0	–1.84 (563)	.07
**Education**						
	Would like to ask questions to health care provider via social media^a^	25.4	23.6	26.6	–0.43 (573)	.67
	Would like to communicate with health care provider via webcam^c^	21.2	18.0	22.9	–0.76 (563)	.45

^a^Estimations for the Dutch population (%) based on survey sample of 581 (54 respondents excluded because they selected no opinion). Note that these estimates are weighted sums of the cell response percentages; therefore, n’s cannot be provided (see Methods) for these percentages.

^b^For gender, group 1=male, group 2=female; for age, group 1=age ≤34 years, group 2=age>34 years; for education, group 1=no or low education, group 2=moderate or high education.

^c^Estimations for the Dutch population based on survey sample of 571 (64 respondents excluded because they selected no opinion). Note that these estimates are weighted sums of the cell response percentages; therefore, n’s cannot be provided (see Methods) for these percentages.

## Discussion

### Principal Findings

As far as we are aware, ours is the first study to investigate online search behavior and preferences regarding the use of social media in health care in the Netherlands. Making use of official statistics, survey results for 635 respondents were successfully extrapolated to the general Dutch population.

The Internet was found to be the number one source for health-related information (82.7%), closely followed by information provided by health care professionals (71.1%). For all groups, the least frequently used source of information was hard copy information, such as leaflets/books. This is higher than AlGhamdi et al [24] found in a survey that included the same age population. They showed that 58.4% of all respondents searched online for health-related information and that health care professionals were the primary source of health-related information. Our findings correspond with a study performed in Brazil, which found that the Internet was the primary source of health-related information for 86% of all respondents [25]. Similar results were also found in a study involving patients suffering from a chronic disease. Approximately 90% of the respondents that searched for additional disease-related information indicated that they used the Internet [26]. However, the same study showed that 55% of all respondents used information leaflets as a source of information versus 14.5% in the present study. This difference can be explained by differences in the study population: our study included any individual instead of patients with a chronic condition only. Another explanation could be that there are differences in broadband penetration between the 2 countries (United States 56.1% vs Netherlands 92.9%) [27]. Health care providers should recognize that a large majority of the Dutch population use online sources for health-related information. Therefore, they should focus on providing high-quality patient information via online channels.

The Dutch population searches online for several health-related topics. In all, 9 of 10 persons indicated that they searched for health-related information at least once a year and 1 in 4 searched for health-related information at least every month. Three topics that were most frequently mentioned (>45.6%) are side effects of medication, symptoms, and diagnoses. People aged 35 years or older searched more often for side effects of medication than their younger counterparts did. This is probably because of a higher consumption of medication by older generations.

Approximately one-third (32.3%) of the Dutch population searches for ratings of health care providers. This is slightly more than was found in a recent report about online health in the United States [28]. This report showed that 10% to 20% of the US population searches for physician ratings, reviews, and rankings. We foresee that more people will search for ratings in the near future, as a rapid rise of health care-related rating websites created by the government, patients’ organizations, and other parties can be witnessed [29]. An example of such a rating site is Zorgkaart Nederland [30], a website containing a database with information about all health care providers in the Netherlands. Anyone can rate their health care provider and add their comments or experiences. Currently, it contains information about 112,832 health care providers. The observation that an increasing number of people share their experiences online is supported by our finding that the Dutch population rates their own opinion as important. Interestingly, patients’ ratings are significantly associated with official patient surveys about the quality of care [31]. This may be an important finding for future researchers and/or governmental parties (eg, health care inspection) because it could help them in determining high-quality care providers, but also in detecting harmful or unwanted situations.

Approximately 1 in 4 persons would like to use social media to consult their physician and 1 in 5 persons would like to communicate with their physician using a webcam. With the growing number of mobile devices, such as smartphones and tablets, we expect the numbers of people wanting to communicate via social media channels or via webcams to increase as well particularly because usability issues for mobile devices are becoming less relevant [32] and there are tools available that use safe connections that protect data and respect the privacy of users, such as Facetalk [33]. Therefore, future researchers should focus on describing best practices for online patient-physician communication and determine the cost-effectiveness. It would also be interesting to study the extent to which face-to-face technology and social media support patient empowerment, which is a term used to describe the process in which consumers are taking an active role in their care process and where the traditional doctor-patient relationship is disappearing [34].

### Limitations

Our study has some limitations that need to be discussed. Although using a social network was helpful in reaching a large group of people very quickly and at relatively low cost, there are some relevant downsides. The online system that sent invitations to Hyves’ members randomly did not allow us to register the number of invitations sent. Furthermore, we were not able to distinguish between people who had actually seen the request but had refused to fill in the survey or people who had not seen the request at all (eg, invitation ended up in spam or junkmail folder). As a result, it was impossible to determine exact response percentages. Although we know that people of all genders, ages, and education levels were active on Hyves at the time of the study and that we corrected for overrepresented or underrepresented groups by using official statistics, it is important to consider that all respondents were recruited via an online social network. As a result, we may have missed a specific subgroup of the Dutch population consisting of people without access to the Internet. However, we believe this group to be small because 92.9% of the Dutch population has Internet access [27]. In relation to the survey, it is important to consider that it did not include questions about diseases and use of medication by respondents, which made it impossible to distinguish between ill and healthy respondents. Realizing that ill patients may have other preferences, future surveys should include questions on this matter. Because the present survey was focused on types of information (eg, social media, Internet, books) future studies should aim to further specify this. For example, they should study which types of social media are used, which search engines are used to search for information, and how consumers rate the reliability of different social media networks or websites.

### Conclusion

The Internet is the main source of health-related information for the Dutch population. One in 4 persons would communicate with their physician via social media channels and it is expected that this number will further increase. Therefore, health care providers should explore new ways of communicating online and should facilitate ways for patients to connect with them. Future research should aim at comparing different patient groups and diseases, describing best practices, and determining cost-effectiveness.

## Multimedia Appendix 1

Survey (English version).

## Abbreviations

CBS: Centraal Bureau voor de Statistiek (Statistics Netherlands)
