# Supplementary material for: Internet and Social Media For Health-Related Information and Communication in Health Care: Preferences of the Dutch General Population
Source: J Med Internet Res. 2013 Oct 2;15(10):e220. doi: 10.2196/jmir.2607 (PMC3806385; doi:10.2196/jmir.2607)
Supplement: Supplementary file 1 [file jmir_v15i10e220_app1.pdf]

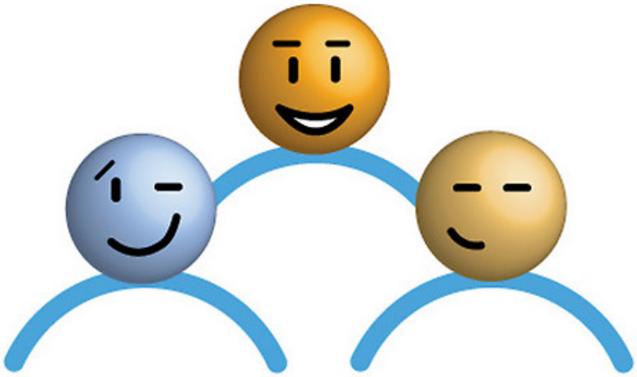

Survey on the use of the Internet and social media for health-related purposes

Hyves

UMC St Radboud

Radboud REshape & Innovation Center

Dear Hyver,

The Radboud REshape & Innovation Center is interested in the extent to which people use the Internet and social media in relation to their health and for health care. If we could achieve a better insight into their preferences and needs, we will be able to adapt health care to it. By participating in this survey you will not only help us, but also yourself.

This survey contains 17 multiple choice questions and it will take approximately 5 minutes of your time.

Thank you very much for your cooperation!

1. Where do you find information about your health? (multiple answers possible)

   ☐ Internet (computer/smartphone e.d.)

   ☐ Physician (e.g. GP)

   ☐ Information leaflets, books

   ☐ Family, friends, or other acquaintances

   ☐ Other: [text box]

2. What do you look for on the Internet? (multiple answers possible)

   Information about:

- ☐ Health care insurance
- ☐ Second opinion
- ☐ Medication and/or side effects
- ☐ Manufacturers of medication (Pharmacy)
- ☐ My hospital or my physician (e.g. GP)
- ☐ Other patients' experiences
- ☐ Specific diagnoses or diseases
- ☐ Therapy or treatment
- ☐ Symptoms
- ☐ Health problems
- ☐ Iets anders, Other, namely:

3. Have you ever searched for ratings of your hospital or your physician?
   - Yes
   - No

4. How often (on average) do you search for health-related information online?

   - Daily
   - > Weekly
   - > Monthly
   - > Annually
   - Never

5. Do you search online **before** visiting your physician (e.g. GP)?
   - Never
   - Rarely

- Sometimes
- Often
- Very Often

6. Do you search online **<u>after</u>** having visited your physician (e.g. GP)?

   - Never
   - Rarely
   - Sometimes
   - Often
   - Very Often

7. How reliable is the information that you find online? [Select on a scale (1-10)]

   Very unreliable 1 2 3 4 5 6 7 8 9 10 Very reliable

How do you perceive the reliability of peoples' advice regarding health-related decisions? [select on a scale 1-10]

Advice from:

8. <u>Your physician (e.g. GP)</u>

   Very unreliable 1 2 3 4 5 6 7 8 9 10 Very reliable

9. <u>Friends or family</u>

   Very unreliable 1 2 3 4 5 6 7 8 9 10 Very reliable

   <u>People on social networks (e.g. Hyves, Facebook)</u>

   Very unreliable 1 2 3 4 5 6 7 8 9 10 Very reliable

   Your own opinion/feeling

   Very unreliable 1 2 3 4 5 6 7 8 9 10 Very reliable

10. Would you like to use a social network (e.g. Hyves, Twitter) to get in touch with your physician (e.g. GP) to be able to ask health-related questions? (If preferred, via a secured connection).
    - Yes

- No
- No opinion

11. Would you like to get in touch with your physician (e.g. GP) or hospital using a webcam?
    - Yes
    - No
    - No opinion

    This an anonymous survey. To correctly interpret the results, we ask for a little more information:

12. Your age: ….

13. Your gender: ☐ male ☐ female

14. Please select your highest level of education

    ☐ No education

    ☐ Lagere school/basisonderwijs (primary education)

    ☐ LBO, VBO, LTS, LHNO, VMBO (Lower vocational education)

    ☐ MAVO, VMBO-t, MBO-kort (Lower general secondary education)

    ☐ MBO, MTS, MEAO (Intermediate vocational education)

    ☐ HAVO, VWO, Gymnasium (Highschool)

    ☐ HBO, HEAO, PABO, HTS (Higher vocational education)

    ☐ Universiteit (Academic level)

    ☐ Other: …. [please specify]

15. First four numbers of your postal code:…..

Thank you!
